# Influence of egg size and parental genetics on the metabolic rate of Chinook and pink salmon embryos

**DOI:** 10.1093/conphys/coaf062

**Published:** 2025-08-20

**Authors:** Alexander T Iritani, Evan M Barnes, Michael P Phelps

**Affiliations:** Department of Animal Sciences, Washington State University, PO Box 647620, Pullman, WA 99164-7620, USA; Department of Animal Sciences, Washington State University, PO Box 647620, Pullman, WA 99164-7620, USA; Department of Animal Sciences, Washington State University, PO Box 647620, Pullman, WA 99164-7620, USA

**Keywords:** Chinook salmon, egg size, metabolism, pink salmon

## Abstract

Freshwater environments are experiencing rapid changes in seasonal temperature and water flows that could impact threatened aquatic species. Environmental stressors experienced by mothers can influence the size and quality of fish eggs creating downstream effects on larval fitness. Cool water fish species like Pacific salmon with extended periods of embryonic development may be especially vulnerable to changing environmental conditions. To gain insight into the factors influencing embryonic physiology in fish, the relationship between parental genetics, egg size and embryo metabolism was examined in developing Chinook salmon (*Oncorhyncus tshawytcha*) and pink salmon (*Oncorhyncus gorbuscha*) embryos, as these species exhibit distinct differences in egg size and life history strategies. Egg size was found to have a relatively limited effect on embryo metabolism with parental genetics having a larger effect on the embryos of these species. Maternal genetics influenced embryonic metabolic rate more in the early stages of development than at later stages of development. These findings suggest that parental genetics or epigenetics is a key factor determining the metabolic rates of salmon embryos and that genetics should be considered when seeking to understand how environmental change will impact threatened fish species, like Pacific salmon.

## Abbreviations

TCSPG: Thorgaard Center for Salmonid Physiology and Genomics
MO2: Oxygen Consumption Rate
RRC: Red Rock Coulee Creek
PRH: Priest Rapids Hatchery
ATU: Accumulated Thermal Units

## Introduction

In egg-laying fish species, a tradeoff exists between the production of numerous small eggs, or less abundant, larger, nutrient-rich eggs. Larger eggs provide more nutrition to the developing embryo, enabling greater development and size of the larval fish before first feeding. Egg size is known to influence larval survival, the development of the embryo, as well as the body size of fry (Allen, 1958; Fowler, 1972; Beacham and Murray, 1985, 1990; Einum and Fleming, 1999; Régnier *et al.,* 2013; Thorn and Morbey, 2017; Leblanc *et al.,* 2022; Yamamoto and Kitanishi, 2022). The influence of egg size on the larval life stage is particularly important, as the larval stage is a critical period commonly associated with poor survival (Kamler, 2008). For species like Pacific salmon that make a significant contribution to egg quality by producing large, nutrient-rich eggs, that support larval development, there is significant variation in egg size between individuals that reflects the maternal investment trade-offs faced by mothers. Variations in the mass of individual salmon eggs can be as high as 300% in some salmon populations (Rombough, 1985). This is likely due to several factors, including maternal size and the environmental conditions experienced during reproductive development. For example, in years when their upstream migration was challenged by high water discharges, female Sockeye salmon that reached the spawning streams invested less in gonads by producing smaller but not fewer eggs (Braun *et al.,* 2013).

The success of fish species like salmon has been thought to be the result of a portfolio of life history strategies that may buffer populations from environmental change by providing varying opportunities to complete their life cycle (Van Den Berghe and Gross, 1989; Acornley, 1999). Salmon populations in the Pacific Northwest are being increasingly threatened due to both natural and anthropogenic factors. Climate models predict a decrease in average stream flow up to 5.5%, and an increase in average river temperature up to 1.68°C in 2080, with an increase in summer water temperature up to 2.1°C in that same time period (Wu *et al.,* 2012). Understanding the mechanisms that contribute to the resiliency of Pacific salmon populations, such as the factors influencing egg size and embryonic physiology in the context of environmental change, is of great importance in developing management strategies to slow population decline (Carvalho, 1993; Shizhen *et al.,* 2002; Braun *et al.,* 2013). For example, in the North Pacific Ocean, there has been a significant decrease in the size and age of maturity in many salmon stocks (Oke *et al.,* 2020). The decreased adult size is a conservation concern for many reasons, including the potential for smaller females to have lower fecundity or reduced egg size (Bilton *et al.,* 1982; Braun *et al.,* 2013).

To investigate how egg size and parental genetics contribute to fish embryo development, we used Pacific salmon embryos as a model species because of their high variation in egg size and long embryonic development phase. We examined the relationship between metabolic rate and egg size across families of both Chinook salmon and pink salmon, which exhibit very different life histories and are the largest and smallest of the Pacific salmon species, respectively. The study focused on the relationship between egg size and embryo metabolism as a measure of embryo growth and health in these different species. The study also used strategic crosses to examine the influence of parental genetics on two stages of embryo development in each species. The findings of our research provide insights into the complex relationship between parental genetics, egg size, and embryo metabolism to gain a more detailed picture of the factors that contribute to embryo health in a threatened species.

## Materials and Methods

### Experimental animals

**Figure 1:**
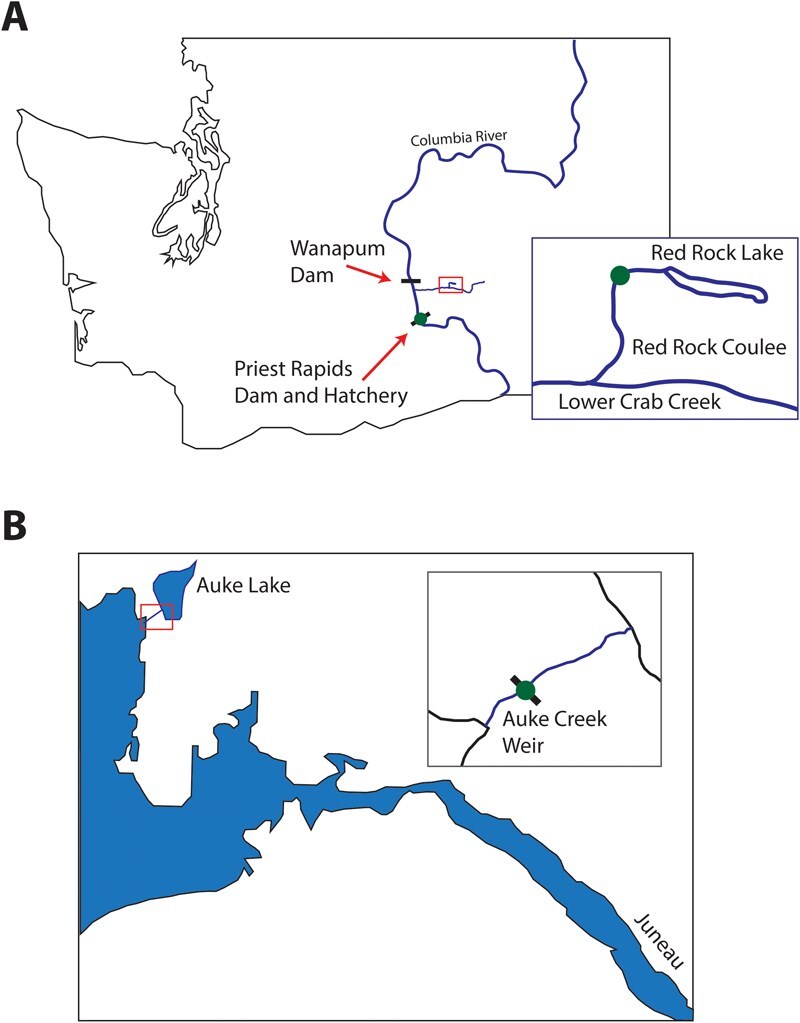
Chinook and pink salmon sampling sites. Illustrated maps of the (A) Chinook salmon sampling sites at Red Rock Coulee and Priest Rapids Hatchery, as well as (B) the Auke Creek weir used for pink salmon collection.

Fall-run Chinook salmon gametes were collected from six female and male spawning adults from the Red Rock Coulee Creek (RRC), a tributary of Lower Crab Creek, which flows into the Columbia River in Central Washington State, USA (Fig. 1A). Fall-run Chinook in Washington State are a run of Chinook that return to spawn in their natal rivers in the fall. They typically spawn shortly after they enter the rivers, as opposed to Spring-run and Summer-run Chinook, which often stage in rivers for months before spawning in the late summer and fall. Sampling of RRC Chinook occurred from 25 to 29 October 2021. This population of fall Chinook is of natural origin and inhabits a stream with unusually warm summer water temperatures (Small *et al.,* 2011). These fish were collected using rod and reel, and approximately 200 eggs from each female and milt from males were manually stripped from the fish prior to releasing them to spawn naturally. Eggs and milt were collected in separate bags containing pure oxygen, and labelled with parental identification numbers. Each fish was sexed, fork length was measured, and a small hole punch was taken from the gill plate to prevent resampling fish. Four hatchery fall-run Chinook salmon females and males were also collected from Priest Rapids Hatchery (PRH) located on the Columbia River downstream of Lower Crab Creek on 6 November 2023 (Fig. 1A). Hatchery-raised salmon have been known to have differing levels of fitness and expression of life-history traits compared to wild fish. Pink salmon gametes from six males and six females were collected from the Auke Creek weir (Juneau, Alaska, USA) during their return migration and spawned on 6 September 2022 (Fig. 1B). Sex and fork length were recorded for each fish. The research station on Auke Creek is jointly operated by the NOAA Alaska Fisheries Science Center, the Alaska Department of Fish and Game (ADF&G), the University of Alaska Fairbanks (UAF), and the University of Alaska Southeast (UAS). The eggs and milt were transported to the Washington State University, Thorgaard Center for Salmonid Physiology and Genomics (TCSPG) hatchery facility, located in Pullman, WA, USA for fertilization. All experimental procedures on animals were approved by the Washington State University, Institutional Animal Care and Use Committee under protocol number 6607. Scientific collection permits were obtained for Chinook salmon from the Washington State Department of Fish and Wildlife (WDFW; # PHELPS 20-255) and pink salmon from the ADF&G (# SF2022-182).

### Measuring egg size and hatching time

**Figure 2:**
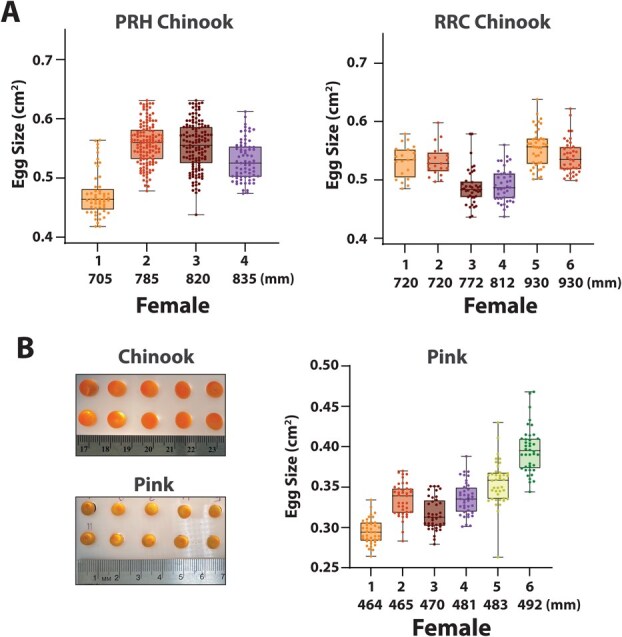
Maternal egg size distribution in Chinook and pink salmon. (A) The size distribution of three-day post-fertilization embryos obtained from fall-run Priest Rapids Hatchery (PRH) Chinook and natural-origin Red Rock Coulee (RRC) Creek Chinook. (B) Images of Chinook and pink salmon eggs during measurement as well as the range of egg sizes found in natural-origin Auke Creek pink salmon. The Chinook salmon and pink salmon egg sizes are ordered in size from smallest (left) to largest (right) fish.

Fall-run Chinook and pink salmon eggs (PRH, RRC and Auke Creek) were measured three days post-fertilization using digital image analysis (Fig. 2). Egg size was measured for four PRH (Hatchery) and six RRC (Natural origin) Chinook salmon families derived from independent females (Fig. 2A), as well as six families of pink salmon (Supplementary material). A family is defined as a set of eggs with unique parental gametes. The number of families analysed for egg size was determined by the number of samples collected for each species and available rearing space. For each family, images were collected for 20 to 40 eggs in a single image. The methods for measuring egg size were adapted from previous studies (Leblanc *et al.,* 2022). Briefly, the eggs were transferred to a white acrylic board containing holes to immobilize the eggs (Fig. 2B). A cell phone camera and an external light source were used to collect images of the eggs for image analysis. A ruler was located next to the embryos in the photo and was used to set the scale for ImageJ software analysis (Schneider *et al.,* 2012). The area of the eggs was determined using the polygon function of ImageJ software (Schneider *et al.,* 2012; cm^2^).

**Figure 3:**
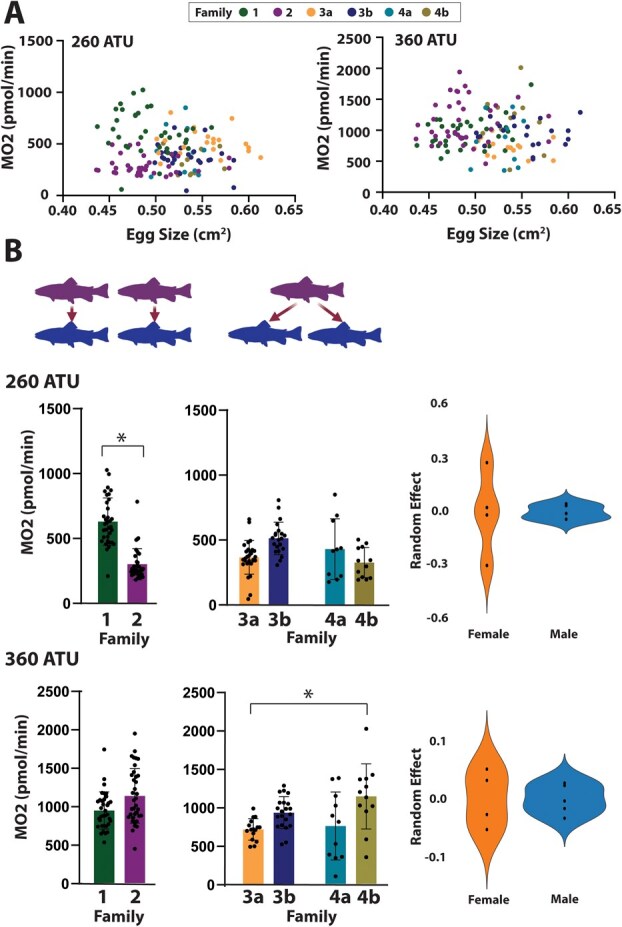
Comparison of egg size and metabolic rate in Chinook salmon embryos. (A) The relationship between egg size and metabolic rate of embryos for six families of RRC Chinook salmon at 260 and 360 accumulated thermal units (ATU) of development. (B) Comparison of the metabolic rate between families with independent female and male parents as well as families sharing a mother with two different fathers. The metabolic rate of the families was compared at 260 ATU and 360 ATU of development. Statistical differences between groups are identified by brackets and asterisk. Visualization of the contribution of mother and father genetics as a random effect are shown with violin plots.

### Oxygen consumption

**Figure 4:**
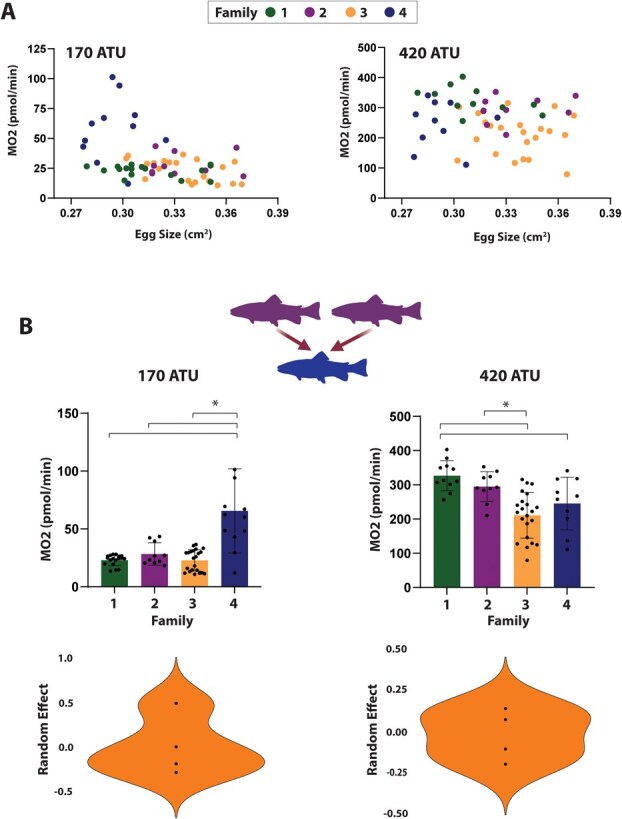
Metabolic rate and egg size relationship in pink salmon embryos. (A) The metabolic rate of four families of pink salmon embryos in relation to their egg size at 170 and 420 ATU of development. (B) Comparison between the families with different females but the same paternal genetics at 170 and 420 ATUs of development. Violin plots show the contribution of maternal genetics to variation in the metabolic rate of the embryos. Statistical differences are identified with an asterisk with the family differences designated by brackets.

For respirometry studies, RRC Chinook salmon families were made by either fertilizing eggs from one female with one male (*n* = 2), or by splitting the eggs from one female and fertilizing the eggs with milt from two different males (*n* = 4; families 3a/b and 4a/b, Fig. 3). These latter crosses were made to examine the influence of paternal genetics on embryonic metabolic rate. Four families of pink salmon were made for respirometry experiments by fertilizing the eggs of four different females with the milt of a single male to isolate the maternal genetic contribution to the embryos (Fig. 4). All embryos were raised in vertical tray incubators in custom incubation boxes containing individual wells for each egg, to separate and independently identify each egg throughout the experiment. For respirometry measurements, different Loligo Systems (Viborg, DK) microplate well sizes were used for Chinook salmon (940 μl) and pink salmon (500 μl) embryos to minimize excess water volume. The optical oxygen sensors present in each well were calibrated with air-equilibrated saturated water and NA_2_SO_3_ treated desaturated water at atmospheric pressure, using the MicroResp™ software (Loligo Systems). The respirometry system was set at 10°C, the same temperature used for embryo incubation. Each microrespirometry well was filled half full of incubator water using a dropper. A single egg was placed in each well and up to 20 eggs were run per plate, with four water-only wells used to normalize background oxygen consumption rates. The wells were then filled to capacity with water and sealed with film, eliminating any air bubbles. The plates were placed into the respirometry chamber for 20 min to measure the rate of oxygen consumption (pmol/min; Polymeropoulos *et al.*, 2016). Mass-specific oxygen consumption rates (MO_2_) were calculated using the MicroResp™ program. MO_2_ calculations are based on the slope of oxygen consumption rate over time, the volume of water in the well and the incubation time.

After collecting all measurements, the eggs were returned to their individual wells in the incubator using egg forceps designed for safe handling of eggs. Respiration rates were standardized to the embryo developmental stage by determining the accumulated thermal units (ATU) at each sampling point. Accumulated thermal units refer to the temperature at which the embryos were incubated (10 C) multiplied by the days post-fertilization. Respiration was measured at 260 and 360 ATU for Chinook salmon and 170 and 420 ATU for pink salmon embryos. The two time points were collected to look at different stages of development, but the timing of sampling was not chosen to analyse any specific developmental stage.

### Statistical analysis

#### Egg size

Normality was assessed on both maternal length and egg size data using the Shapiro–Wilk test in R version 4.4.1 (R Core Team, 2021). Linear mixed models (LMM) with random effects were assessed on the maternal length and egg size independently for both Chinook salmon populations (PRH and RRC), as well as pink salmon species, with maternal length as the predictor variable and maternal genetics as the random effect using the ‘lmer’ function from the ‘lmertest’ package (version 3.1-3; Kuznetsova *et al.,* 2017). A one-way ANOVA was performed using GraphPad Prism software 10.4.1 for mac (Boston, MA) to determine differences in egg size for each Chinook salmon (PRH, and RRC) and pink salmon (Auke Bay) population, comparing the size of eggs derived from each family within each population. Tukey's multiple comparison tests were performed to identify differences between the populations of fall-run Chinook salmon. Variation in egg size between populations was confirmed to be equal using an *F*-test.

#### Metabolic rate

For oxygen consumption analysis, a Shapiro–Wilk test was performed to assess normality and it was discovered that the MO_2_ data was not normally distributed. A Kruskal–Wallis test was performed on the respirometry data from Chinook and pink salmon embryos using the ‘base R’ package in R, with Tukey's multiple comparison tests to determine statistical differences between families with the level of significance set at *P* = 0.05. To assess the influence of parental genetics on embryo metabolism a generalized linear mixed model (GLMM) was used with a negative binomial distribution to account for overdispersion. The GLMM was calculated using the ‘glmer’ function from the ‘lme4’ package (version 1.1-3.6; Bates *et al.,* 2015) in R to determine the effects of egg size and maternal and paternal identities as random effects on the metabolic rate. Egg size was the fixed effect and males and females were analysed as random effects in explaining variation in MO_2_ values. Akaike Information Criterion (AIC) was analysed on both the GLMM including parental random effects, and without the parental random effects. The model which included the lowest AIC value was used. Because one male was used for all pink salmon families, the analysis consisted of egg size as the fixed effect and females as the only random variable.

## Results

### Maternal effect on egg size across salmon populations and species

Egg size in fall-run Chinook from RRC and PRH varied significantly between females (*P* < 0.0001; PRH, 0.54 ± SE 0.0024; RRC, 0.53 ± SE 0.0022 cm^2^). In Priest Rapids fish, a statistically significant relationship was found between egg size and maternal length (PRH: [β, 0.00062 ± 0.00020 SE; *t*, 3.19; *P* = 0.081). The random effect of maternal ID in our LMM showed small contribution in being able to explain variation (σ^2^, 0.00035) relative to the residual variance (σ^2^, 0.00221). In Red Rock Coulee fish, **maternal length did not significantly predict egg size** (β, 0.00015 ± 0.00031 SE; *t*, 0.485; *P* = 0.661). The random effect of Female ID in the LMM accounted for more variation (σ^2^, 0.00225) than the residual error (σ^2^, 0.00100). There was a statistically significant relationship between egg size and maternal length in pink salmon, with larger females having larger eggs (*R*^2^ = 0.7149; *P* = 0.0184; Fig. 2B). The eggs collected from Auke Creek pink salmon were on average 63% smaller than the size of Chinook salmon eggs (Pink; 0.34 cm^2^ ± 0.0035 SE, Chinook; 0.53 cm^2^ ± 0.0053 SE; Fig. 2B). The variation in egg size within each female pink salmon was also lower than Chinook salmon (*P* < 0.001).

### Parental genetics, not egg size, influences the metabolic rate of salmon embryos.

There was a significant difference in MO_2_ between families of Chinook salmon at both 260 and 360 ATU of development (Kruskal–Wallis: *P* < 0.0001 and *P* < 0.0001, respectively; Fig. 3A, B). At 260 ATU of Chinook salmon development, statistical differences were observed in fish with independent maternal and paternal genetics (cross 1 and 2, 1 and 3b, 1 and 4b, 2 and 3a; Fig. 3B). Egg size was not found to significantly influence MO2 (β, −0.4922; *P* = 0.673, SE:1.1667). Inclusion of parental random effects improved model fit, as evidenced by lower AIC (1725.98) compared to the model excluding parental effects (AIC:1793.29). Maternal effect contributed to more variability than paternal effect at this developmental stage (female σ^2^, 0.0508; male σ^2^, 0.0067; Fig. 3). Lower AIC indicates a better model fit and whether inclusion of the random effect is appropriate. At 360 ATU of Chinook salmon development there was a slight negative relationship of egg size on MO_2_ (β, −1.942; *P* = 0.028, SE: 0.8810). It was also found that genetics did not improve the model fit (AIC with parental genetics: 1858.407, AIC without parental genetics, 1854.036). Differences in metabolism were detected between independent parents (cross 2 and 3a; *P* = 0.001) as well as in crosses with shared maternal genetics but different paternal genetics at 360 ATU (cross 3a and 4b; p:0.02; Fig. 3B).

Significant differences were detected between pink salmon families sharing the same paternal genetics but different maternal genetics at 170 and 420 ATU of embryonic development (Kruskal–Wallis test, 170 ATU: *P* = 0.0002, 420 ATU: *P* < 0.0001; Fig. 4B). In pink salmon embryos at 170 ATU, we found that egg size had a significant negative relationship with MO_2_ (eff. size: −5.095; *P* = 0.043; SE: 2.515) but this was not the case at 420 ATU (β, −1.241; *P* = 0.508; SE: 1.874; Fig. 4). Inclusion of the maternal effect improved model fit in both 170 (AIC with parental effect, 472.7806, AIC without parental effect: 493.5023; female σ^2^, 0.093) and in 420 ATU but to a lesser degree (AIC with parental genetics: 615.9991, AIC without parental genetics: 618.2920; female σ^2^, 0.023).

### Embryonic oxygen consumption between Chinook and pink salmon

**Figure 5:**
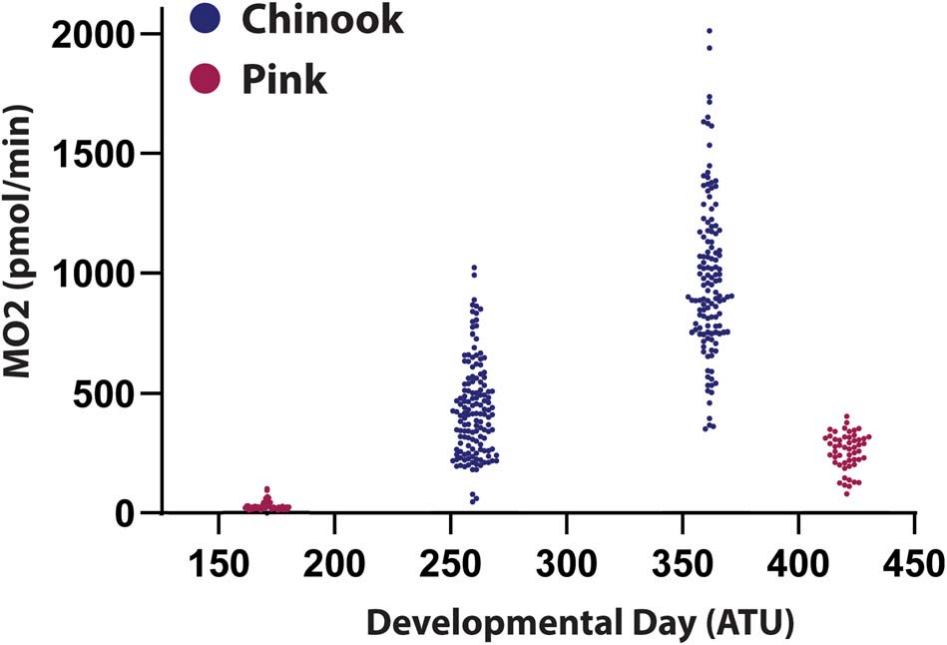
Changes in metabolic rate of Chinook and pink salmon during embryonic development. The change in MO_2_ of Chinook salmon (260 ATU and 360 ATU) and pink salmon (170 ATU and 420 ATU) embryos at two developmental time points.

Given that egg and offspring size vary between Chinook and pink salmon, differences in the rate of change in oxygen consumption throughout development were compared between the two species. The change in metabolic rate during embryonic development was higher for Chinook salmon than pink salmon even though pink salmon were measured at a more advanced stage of development (*P* < 0.0001; Fig. 5).

## Discussion

In this study, we observed significant variation in egg size between females with Chinook salmon having higher variation in egg size compared to pink salmon (Fig. 2). While significant relationships were found between female size and egg size in pink salmon, we did not observe a similar relationship with our Chinook salmon samples. However, to accurately determine the relationship between egg size and female size in our salmon populations would have required a significantly larger sample size and a different experimental design than was conducted in this study. Egg size has been shown to be positively correlated with maternal body size in Pacific salmonids (Kindsvater *et al.,* 2016). A similar trend has also been seen in Atlantic salmon, where, on average, larger eggs are produced from larger mothers (Thorpe *et al.,* 1984). In contrast to these studies, some research has failed to establish a clear correlation between egg size and maternal body length (Dickerson *et al.,* 2005), suggesting that egg size may be independent of female size under some conditions. Evidence suggests that larger females either have larger or more numerous eggs as they have more energetic resources to allocate to their offspring (Beacham and Murray, 1993). Populations of salmon with difficult and/or lengthy migrations have fewer and smaller eggs, with fecundity being highly correlated to migration length (Fleming and Gross, 1989, 1990; Kinnison *et al.,* 2001). However, selection for egg size versus fecundity likely differs based on the local environmental conditions experienced by specific salmon species and populations (Rollinson and Hutchings, 2013).

There is some controversy over the importance of egg size in salmon resource management. Some studies support a ‘bigger is better’ hypothesis, which proposes that fitness increases with increasing egg size (Smith and Fretwell, 1974; McGinley *et al.,* 1987; Sargent *et al.,* 1987). In Atlantic salmon, increasing egg size resulted in lower metabolic demand because the greater surface area on larger eggs may be more efficient at extracting oxygen (Einum and Fleming, 2002; Hendry and Day, 2003; Rombough, 2007). Other studies suggest that under lower dissolved oxygen conditions, larger eggs have lower survival (Hendry *et al.,* 2001; Kinnison *et al.,* 2001), while others have found that larger eggs survived better in lower dissolved oxygen environments (Einum and Fleming, 2002; Rombough, 2007). We observed that Chinook salmon have a significantly higher change in metabolic rate between early and late embryonic stages, suggesting that the larger size of Chinook salmon larvae is not just due to the extended length of embryonic development but that the growth rate of the embryo is faster in this species (Fig. 5). The fact that Chinook salmon eggs are significantly larger than pink salmon eggs and have a higher metabolic rate, emphasizes that the relationship between egg size and embryo metabolism is species-specific.

There is an alarming decline in the size and age of many salmon species, particularly in hatchery-reared fish, the causes of which are not fully understood (Ricker, 1981; Oke *et al.,* 2020). One concern is that smaller returning females will produce smaller eggs, which may lead to a reduction in the fitness of the offspring. We found that both parental genetics and egg size contributed to variation in the metabolic rate of offspring. This effect was common to both Chinook and pink salmon species. A negative relationship between egg size and metabolism was observed in both species, but the effect was limited and it was not identified at all of the developmental time points analysed in the study. By examining numerous eggs from each family, we showed that there is high within-family variation in the metabolic rate of salmon embryos, which could support the theory that salmon embryos differ in their metabolic demands. This may provide more overall resilience to environmental fluctuations than embryos with a narrow physiological range. Increasing the number of families analysed in future studies at the expense of family egg numbers could provide stronger support for the between-family observation that parental genetics has a strong role in driving the metabolic rate of salmon embryos. It is clear from our findings that parental genetics should be an important component of studies of embryonic development and fitness. While we described differences between families as genetic effects, variations in the metabolic rate between families could be the result of both the genetic and/or epigenetic contributions of the parents. There is a growing appreciation for the strength of epigenetic alterations on salmon life history decisions (Åsheim *et al.,* 2023). Epigenetic modifications driven by environmental cues occur on shorter timeframes than inherited genetic changes and can be passed from parent to offspring (i.e. transgenerational epigenetic inheritance; Thompson and Blouin, 2015; Ryu *et al.,* 2018; Anastasiadi *et al.,* 2021).

After separating out maternal and paternal effects, we observed that the effect of maternal genetics in Chinook salmon was greater earlier in development than the paternal effect, but as the embryo developed, both the maternal and paternal effects attributed to similar amounts of variation in MO_2_ (Fig. 3B). In pink salmon, although the paternal effect was not experimentally tested, the influence of maternal genetics was identified at 170 ATU and less so at 420 ATU, which is consistent with our Chinook salmon data. This suggests that early in development, embryonic metabolism may be influenced by maternal genetics, while later in development the genetics of the embryo (i.e. the contribution of both mother and father) may play a greater role in determining the metabolic rate. While not specifically addressed in our research, it is possible that the maternal influence on early salmon embryos is the result of maternal RNAs established during oogenesis. If further research establishes this link, then environmental conditions experienced by the mother that alter maternal RNA composition could have downstream impacts on early embryo metabolism. The inherent variability in embryonic metabolism that we observed between families may be a common feature in Pacific salmon, which provides the flexibility necessary to promote successful recruitment even in unpredictable stream conditions. Understanding how changing environmental conditions will impact egg quality and embryo development will be important in guiding management practices to protect threatened species such as Pacific salmon.

## Supplementary Material

Web_Material_coaf062

## Data Availability

All data from the manuscript can be found in the supplemental data files.
